# Standardization of the liquid biopsy for pediatric diffuse midline glioma using ddPCR

**DOI:** 10.1038/s41598-021-84513-1

**Published:** 2021-03-03

**Authors:** Daphne Li, Erin R. Bonner, Kyle Wierzbicki, Eshini Panditharatna, Tina Huang, Rishi Lulla, Sabine Mueller, Carl Koschmann, Javad Nazarian, Amanda M. Saratsis

**Affiliations:** 1 Department of Neurological Surgery, Loyola University Medical Center, Maywood, IL USA; 2 Center for Genetic Medicine Research, Children’s National Medical Center, Washington, DC USA; 3 The George Washington University School of Medicine and Health Sciences, Washington, DC USA; 4 Department of Pediatric Hematology/Oncology, University of Michigan Medical Center, Ann Arbor, MI USA; 5 Department of Pediatric Oncology, Dana-Farber Cancer Institute, Boston, MA USA; 6 Department of Neurological Surgery, Northwestern University Feinberg School of Medicine, Chicago, IL USA; 7 Department of Pediatric Hematology/Oncology, Brown Alpert Medical School, Providence, Rhode Island USA; 8 Department of Neurology, University of California San Francisco, San Francisco, CA USA; 9 Department of Oncology, Children’s Research Center, Diffuse Midline Glioma (DMG) Research Center, University Children’s Hospital Zürich, Zürich, Switzerland; 10 Division of Pediatric Neurosurgery, Department of Surgery, Ann & Robert H. Lurie Children’s Hospital of Chicago, 225 E Chicago Ave Box 28, Chicago, IL 60614 USA; 11 Department of Biochemistry and Molecular Genetics, Northwestern University Feinberg School of Medicine, Chicago, IL USA; 12 The Brain Tumor Institute, Children’s National Health System, Washington, DC USA

**Keywords:** Cancer genetics, CNS cancer, Tumour biomarkers

## Abstract

Diffuse midline glioma (DMG) is a highly morbid pediatric brain tumor. Up to 80% of DMGs harbor mutations in histone H3-encoding genes, associated with poor prognosis. We previously showed the feasibility of detecting H3 mutations in circulating tumor DNA (ctDNA) in the liquid biome of children diagnosed with DMG. However, detection of low levels of ctDNA is highly dependent on platform sensitivity and sample type. To address this, we optimized ctDNA detection sensitivity and specificity across two commonly used digital droplet PCR (ddPCR) platforms (RainDance and BioRad), and validated methods for detecting *H3F3A* c.83A > T (H3.3K27M) mutations in DMG CSF, plasma, and primary tumor specimens across three different institutions. DNA was extracted from H3.3K27M mutant and H3 wildtype (H3WT) specimens, including H3.3K27M tumor tissue (n = 4), CSF (n = 6), plasma (n = 4), and human primary pediatric glioma cells (H3.3K27M, n = 2; H3WT, n = 1). ctDNA detection was enhanced via PCR pre-amplification and use of distinct custom primers and fluorescent LNA probes for c.83 A > T *H3F3A* mutation detection. Mutation allelic frequency (MAF) was determined and validated through parallel analysis of matched H3.3K27M tissue specimens (n = 3). We determined technical nuances between ddPCR instruments, and optimized sample preparation and sequencing protocols for H3.3K27M mutation detection and quantification. We observed 100% sensitivity and specificity for mutation detection in matched DMG tissue and CSF across assays, platforms and institutions. ctDNA is reliably and reproducibly detected in the liquid biome using ddPCR, representing a clinically feasible, reproducible, and minimally invasive approach for DMG diagnosis, molecular subtyping and therapeutic monitoring.

## Introduction

Diffuse midline glioma (DMG) is a highly morbid pediatric central nervous system (CNS) tumor for which there is currently no effective treatment. Approximately 20% of pediatric CNS tumors occur in the brainstem, of which up to 80% are DMG^1^. Due to their anatomic location and infiltrative nature, DMGs are not amenable to surgical resection and are most often diagnosed radiographically and treated with radiation therapy, with no effect on survival^2–5^. Recent studies of DMG biology revealed distinct genomic alterations compared to hemispheric pediatric and adult gliomas^2,3^. Specifically, 80% of pediatric DMGs harbor somatic mutations in histone H3-encoding genes *H3F3A* (60%)*, HIST2H3C* or *HIST1H3B/C* (20%)*,* resulting in lysine-27-to-methionine (H3K27M) conversion that confers a more aggressive clinical course and poorer overall response to therapy^6–12^. As such, the World Health Organization (WHO) classified H3K27M mutant DMG as a distinct clinical entity in 2016, with the biological and clinical implications of the H3 mutation making detection critical for diagnosis, treatment, and clinical trial enrollment^13–15^. While clinically feasible, stereotactic tumor tissue biopsy for mutation detection is not without significant surgical risk^16^. Further, tumor response to therapy is typically monitored by serial conventional MRI, making it challenging to discern between pseudo-progression and progressive disease. In contrast, tumor “liquid biopsy” via cerebrospinal fluid (CSF) or blood sampling may represent a more clinically feasible, less invasive approach for evaluating tumor biology and treatment response^17^.

It is crucial for clinicians and scientists to consider new technical approaches as the molecular understanding and treatment of DMG evolves. We previously reported *H3F3A* c.83 A > T (H3.3K27M) mutation detection in circulating tumor DNA (ctDNA) in CSF and plasma of children with DMG, and developed a digital droplet PCR (ddPCR) approach to detect and monitor H3.3K27M mutation in ctDNA from the DMG liquid biome^17–19^. Our work, and mounting evidence in the literature, demonstrate that liquid biopsy is a viable tool for clinicians to diagnose and monitor pediatric CNS tumors^20^. Clinical implementation of this approach requires exquisite test reliability, sensitivity and specificity. However, inter-institutional differences in ddPCR instruments and protocols impede the broad clinical application of this technique. Additionally, poor access to specimens further exacerbates the challenge of validating and optimizing these analytic methods for rare pediatric CNS tumors.

To address these challenges, we optimized our ddPCR-based technique for H3.3K27M detection using matched DMG tissue and liquid biopsy specimens, and validated our approach across three academic institutions using two leading ddPCR instruments. Here, we show high test sensitivity, specificity, and reproducibility for detecting and quantifying H3.3K27M mutant ctDNA across institutions and platforms, which is essential for clinical implementation of this powerful new approach.

## Methods

### Biological specimens

Patient specimens (Supplementary Table 1) were collected during the course of treatment (PNOC003, NCT02274987) or upon autopsy, after informed consent was obtained, as approved by Institutional Review Boards (Lurie Children’s Hospital of Chicago 2012-14877 and 2005-12252, Northwestern University STU00202063, University of California San Francisco 14-13895, University of California San Diego 150450, and Children's National Health System 1339, 747). For subjects under 18, informed consent was provided from a parent or guardian. All patient identifiers were removed with de-identified numerical identifiers assigned. In addition, all methods were performed in accordance with the relevant guidelines and regulations.

CSF from children with brain tumors (n = 5, Table 1) was collected during the course of treatment or at autopsy. CSF from a patient with congenital hydrocephalus was used as negative control. CSF specimens were centrifuged at 500×*g* for five minutes at 4 °C (NU), or at 5000×*g* for 10 min at 4 °C (CN) via established institutional biobanking protocols. The resulting cell-free supernatant was collected, aliquoted, and stored at − 80 °C.

**Table 1:** Custom, sequence specific primers and fluorescent locked nucleic acid (LNA) probe sets utilized.

	Assay A (Pandarithna et al., CN)	Assay B (Stallard et al., UM)	Assay C (Huang et al., NU)
Forward primer	5′-GTACAAAGCAGACTGCCCGCAAAT-3′	5′-GGTAAAGCACCCAGGAAG-3′	5′-TGCTGGTAGGTAAGTAAGGAG-3′
Reverse primer	5′-GTGGATACATACAAGAGAGACTTTGTCCC-3′	5′-CAAGAGAGACTTTGTCCC-3′	5′-CAAGAGAGACTTTGTCCC-3′
Wild-type probe	/5HEX/CA + C + T + C + T + T + GC/3IABkFQ/	5′-HEX-TC + GC + A + A + GA + GT + GC-IABkFQ-3′	n/a (used only primers for pre-amplification)
Mutant probe^a^	/56-FAM/CA + CT + C + **A** + T + GCG/3IABkFQ/	5′-6-FAM-TC + GC + A + **T** + GA + GTGC-IABkFQ-3′	
ddPCR Amplicon	173 bp	130 bp	300 bp

*HEX* hexachlorofluorescein, *6-FAM* 6-carboxyfluorescein, *IABkFQ* Iowa Black FQ quencher.

^a^Mutant base is bold, “ + ” denotes LNA bases.

Plasma or serum was collected from patients with DMG enrolled in the PNOC003 at the time of diagnosis (NCT02274987) or at autopsy (n = 4) (Table 1). Whole blood was collected in purple top potassium EDTA tubes for plasma isolation, inverted and spun at 2000×*g* for 15 min at 4 °C. For serum samples, blood was collected in gel-barrier tubes with clot activator and gel and incubated at room temperature for 30 min. Blood was centrifuged to separate plasma/serum (supernatant), white blood cells, and red blood cell pellet. Plasma/serum was aliquoted into cryovials and stored at − 80 °C.

Tumor tissue was obtained from DMG patients during the course of treatment or at autopsy (n = 4) and stored at − 80 °C. *H3F3A* mutation status was validated via DNA sequencing (Supplementary Table 1). Pediatric glioma cell lines SF7761 (H3.3K27M DMG) and KNS42 (H3.3G34V supratentorial pediatric high-grade glioma, were cultured as previously described and used for analysis (Supplementary Table 1)^12,21,22^.

### DNA extraction

QIAamp DNA Mini Kit (Qiagen) was used to extract genomic DNA (gDNA) from 5 × 10^6^ cells per manufacturer’s protocol. Tumor tissue gDNA was extracted using Gentra Puregene tissue kit (Qiagen) according to manufacturer’s instructions. Cell free DNA (cfDNA) was extracted from 1 mL plasma using the QIAmp Circulating Nucleic Acid Kit (Qiagen) per manufacturer’s protocol. Qiagen protocol for purification of circulating nucleic acids from 1 mL of urine was used to extract ctDNA from 500 µL of CSF. cfDNA was eluted in 100 µL buffer AVE or MB grade H_2_O twice in order to increase DNA yield. Extracted gDNA and cfDNA were quantified using Nanodrop Nucleic Acid Quantification (Thermo Fisher Scientific) and Qubit Fluorometric Quantitation (Thermo Fisher Scientific). The 2100 Bioanalyzer (Agilent Technologies) was used to assess DNA quality of cfDNA extracted from plasma samples (Supplementary Fig. 1).

### DNA PCR Pre-amplification

gDNA extracted from tumor tissue and cells, and cfDNA extracted from CSF and plasma, was pre-amplified at CN using Q5 hot start high-fidelity master mix (New England Biolabs), and at NU using SsoAdvanced PreAmp Supermix (Biorad), with 50 nmol/L each of forward and reverse primer. Pre-amplification at CN was performed using Assay A primers (Supplementary Fig. 1) in ABI 2720 thermocycler: 98 °C for 3 min; nine cycles of 98 °C for 10 s, 58 °C for 3 min, 72 °C for 30 s; and an extension of 72 °C for 2 min. Product was diluted 1:5 with TE buffer (pH 8.0). Pre-amplification at NU was performed on the BioRad T100 thermocycler using the following conditions: 95 °C for 3 min, 10 cycles of 95 °C for 15 s, annealing temperature (58 °C) for 4 min. The pre-amplified product was diluted 1:5 with molecular grade water. At CN, 0.025 ng gDNA from DMG-51-T was used as a positive control per a previously established institutional protocol^17,19^. 2 ng of tumor gDNA was used for ddPCR analysis of patient-matched tumor, CSF and plasma/serum specimens. Where applicable, starting cfDNA aliquots were speed-vacuum concentrated from 100 µL to 10.5–11 µL prior to pre-amplification. Assay A primers were used for ctDNA pre-amplification of all samples at CN, while Assay C primers were used for PCR pre-amplification at NU^18^.

### ddPCR analysis

Custom sequence-specific primers and fluorescent locked nucleic acid (LNA) probes for *H3F3A* amplification and sequencing were used based on previously reported assay designs by collaborating institutions (Table 1, Supplementary Fig. 2)^18,19,23^. ddPCR reactions at CN were conducted using RainDrop according to manufacturer instructions (RainDance Technologies). ddPCR was conducted with 1 × TaqMan Genotyping Mastermix (Life Technologies), 1 × RainDance droplet stabilizer, 12 µL target DNA product, 900 nmol/L forward and reverse primers, and 200 nmol/L mutant and wildtype probes. The following ddPCR protocol was used: 1 cycle at 95 °C for 10 min, 45 cycles at 95 °C for 30 s and 58 °C for 2 min, 1 cycle at 98 °C for 10 min, and 1 cycle at 10 °C infinite, all at a ramp rate of 0.5 °C/s.

ddPCR reactions performed at NU and UM were conducted using BioRad Q200 according to the manufacturer’s instructions. ddPCR reactions were conducted with 2 × ddPCR Supermix for probes (BioRad, no dUTP), 1–5 µL of target DNA product, 900 nmol/L of forward and reverse primers, and 200 nmol/L of mutant and wildtype probes. The following ddPCR protocol was used at UM: 1 cycle at 95 °C for 10 min, 40 cycles at 94 °C for 30 s and 58 °C for 1 min, 1 cycle at 98 °C for 10 min, and 1 cycle at 12 °C infinite, all at a ramp rate of 2 °C/s. Since Assay A was originally designed for the RainDance platform, a modified ddPCR thermocycling protocol was employed to optimize droplet detection and results on the BioRad platform at NU. The ddPCR protocol employed was: 1 cycle at 95 °C for 10 min, 45 cycles at 95 °C for 30 s and 58 °C for 2 min, 1 cycle at 98 °C for 10 min, and 1 cycle at 10 °C infinite, all at a ramp rate of 2 °C/s. All plasma and CSF samples were analyzed in technical duplicate or triplicate based on sample availability.

### Statistical analysis

Data generated on BioRad Q200X was analyzed with Quantasoft AnalysisPro, while data generated on RainDance was analyzed with RainDrop Analyst II (Supplementary Table 2)^17^. Mutation allelic frequency (MAF) for each sample was calculated as the number positive mutant droplets divided by the sum of the positive mutant and wildtype droplets detected. Poisson-corrected droplet counts were used to calculate MAF. Nonparametric tests were used for results analyses. All data points represent technical duplicates or triplicates based on sample availability. Paired samples were analyzed by Wilcoxon signed-rank test, and unpaired samples were analyzed by Mann–Whitney test. Threshold for false positive droplets was based on non-template control samples analyzed with test samples in each assay; the number of false positive droplets detected in the negative control sample were subtracted from the number of positive droplets detected in the test samples, and thereby accounted for when calculating MAF. For all analyses, a p-value < 0.05 defined statistical significance.

### Ethical approval and consent to participate

All patient specimens were collected during the course of treatment (PNOC003, NCT02274987) or upon autopsy, after informed consent was obtained as approved by the Institutional Review Boards for Ann & Robert H. Lurie Children’s Hospital of Chicago, Northwestern University (Lurie 2012-14877 and 2005-12252, NU STU00202063), the University of California San Francisco (San Francisco, CA; IRB #14-13895), University of California San Diego (San Diego, CA; IRB #150450), and Children's National Health System (IRB #1339, #747). All patient identifiers were removed at the time of specimen collection, with a de-identified numerical identifier assigned to each specimen before processing. Histone mutation status of each subject was confirmed by molecular analysis of tumor tissue.

## Results

### Optimization of ctDNA droplet detection

**Figure 1:**
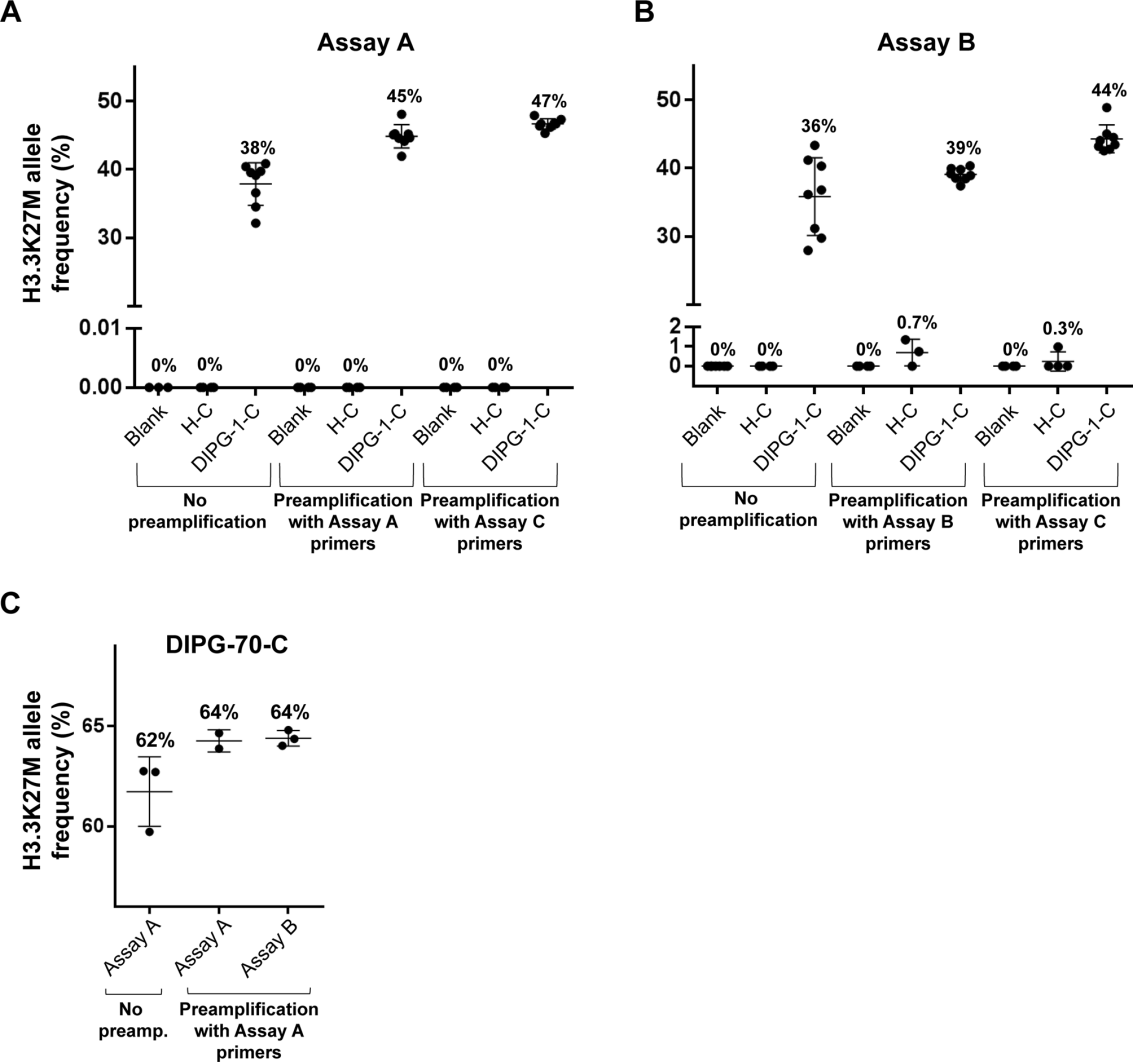
ddPCR analysis of CSF ctDNA across platforms, with and without pre-amplification. The effect of DNA pre-amplification on BioRad platform test sensitivity was evaluated using ctDNA extracted from H3.3K27M mutant CSF (DMG-1-C), non-tumor CSF (H-C) and non-template controls (blank). (**A**) ddPCR with Assay A and (**B**) Assay B, after PCR pre-amplification of input DNA using the same respective primer set resulted in greater droplet counts compared to ddPCR without prior DNA PCR pre-amplification. A further increase in droplet detection was achieved following DNA pre-amplification using a 300 bp *H3F3A* primer (Assay C), with no observed change in detected MAF with either assay after pre-amplification. (**C**) RainDance platform results. The effect of DNA pre-amplification on RainDance platform test sensitivity was evaluated using ctDNA extracted from H3.3K27M mutant CSF (DMG-70-C). PCR pre-amplification of input DNA using Assay A or Assay B increased detected droplet counts, relative to ddPCR analysis alone by Assay A, with no effect on observed MAF. % values = Mutation Allelic Frequency (MAF); * = Statistically significant difference in MAF (t-test*, p* < 0.05).

We compared two PCR primer and probe sets developed at two participating institutions, in order to determine optimal ddPCR reaction conditions (Table 1, Supplementary Fig. 2). For CSF ctDNA analysis, we detected fewer false positive droplets with Assay A on the BioRad platform (Fig. 1A,B), which may be attributed to the shorter length of Assay A probes improving the specificity of probe-target DNA hybridization. No significant difference in CSF droplet counts or MAFs were observed between assays on the RainDance platform (Fig. 1C). On the BioRad platform, Assays A and B resulted in similar droplet counts and MAFs, but A resulted in poorer separation between wildtype, mutant and negative clusters compared to B. This effect was more pronounced following preamplification (Supplementary Fig. 3A), which could be attributed to the longer amplicon length. To address this, gating adjustments were tailored to ensure maintenance of droplet non-detection on non-template control samples, while maximizing positive wildtype droplet detection in target reaction wells. These gating adjustments were applied uniformly across all ddPCR assays run on a single plate. In contrast, distinct separation of negative, wildtype and mutant droplet clusters was achieved using both assays on the RainDance (Supplementary Fig. 3B). False positive droplets did not exceed 5 droplets per sample on the BioRad platform, and fewer false positives were detected on the RainDance platform. Droplet separation was optimized at an annealing temperature of 58 °C (Supplementary Fig. 4A). Increasing thermocycling to 45 PCR cycles improved separation without increasing false positives, and was used for all subsequent ddPCR analyses with Assay A at NU (Supplementary Fig. 4B).

### ctDNA pre-amplification increases ddPCR sensitivity

We tested the effect of ctDNA pre-amplification on ddPCR assay sensitivity (Fig. 1). H3.3K27M mutant (DMG-1-C) and H3WT CSF (H-C) were analyzed with non-template controls on the BioRad platform as follows: (1) no pre-amplification; (2) PCR pre-amplification with primers used for subsequent ddPCR; or (3) PCR pre-amplification with Assay C primers, and subsequent ddPCR with Assay A or B primers (Fig. 1A,B, Supplementary Fig. 3A). We detected greater mutant droplets and MAF values with pre-amplification, regardless of PCR primers used, with no change in test specificity. False positive mutant droplet detection in the H3WT sample was observed after Assay B PCR pre-amplification and ddPCR analysis, but was not statistically significant.

RainDance was used to test similar workflows for H3.3K27M mutant CSF analysis (DMG-70-C): (1) no pre-amplification; or (2) pre-amplification with Assay A followed by ddPCR with either Assay A or B (Fig. 1C, Supplementary Fig. 3B). Again, more mutant droplets and greater MAF values were observed with ctDNA pre-amplification regardless of PCR primers used, with no change in test specificity. ddPCR analysis results were not affected by the specific primers used for PCR pre-amplification, provided that primers used for subsequent ddPCR were identical to, or nested within, the pre-amplification primer amplicon (Table 1, Supplementary Fig. 2).

### Optimizing low ctDNA detection

**Figure 2:**
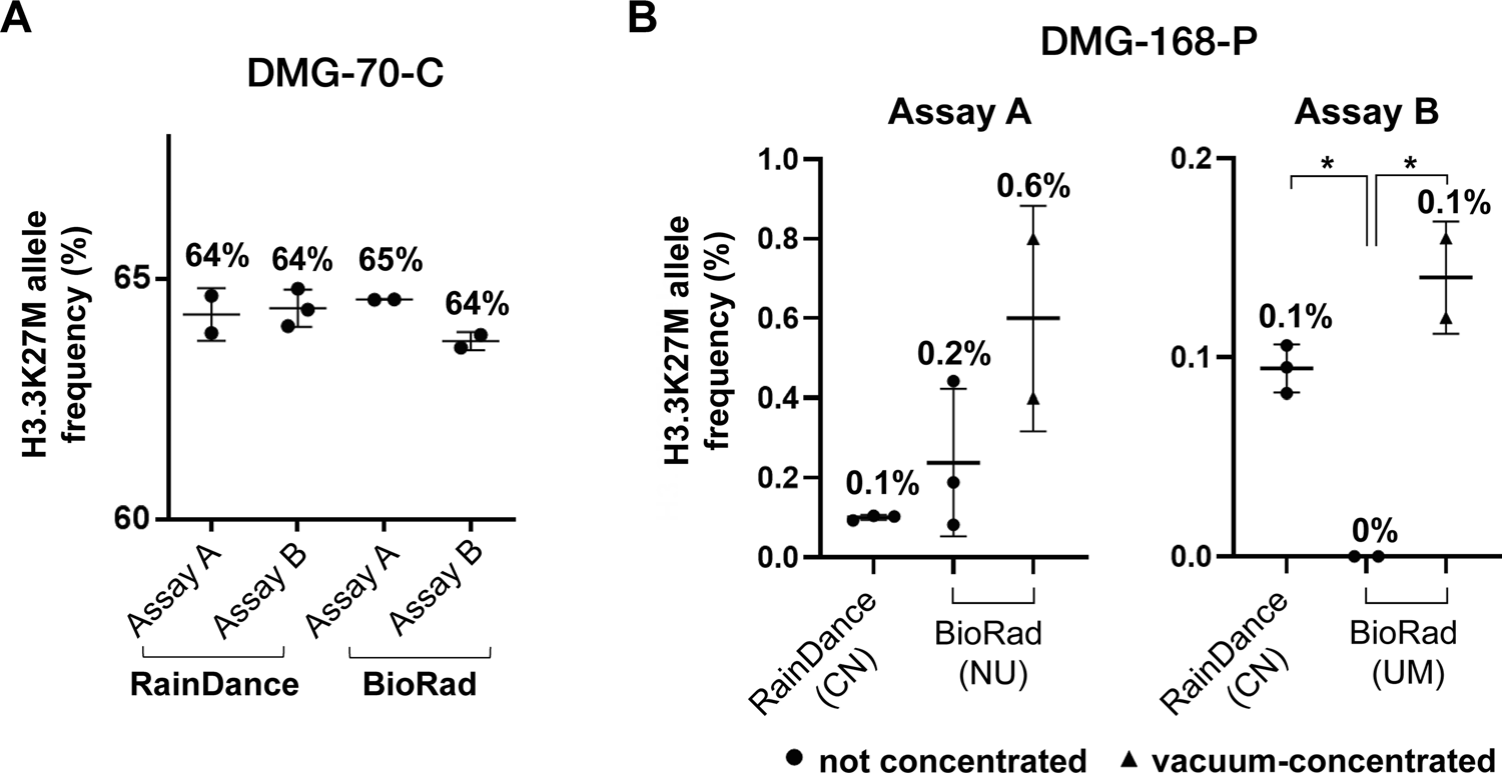
Optimization of ctDNA detection across technical platforms and institutions. ctDNA extracted from CSF (DMG-70-C) and plasma (DMG-168-P) samples were analyzed for H3K27M mutation on BioRad and RainDance platforms at multiple institutions (CN, NU, UM). (**A**) CSF-derived ctDNA ddPCR results. CSF-derived ctDNA was pre-amplified at CN prior to ddPCR analysis. A 12 µL of sample was analyzed on the RainDance platform (CN), and 1 µL analyzed on the BioRad platform (UM). Fewer positive droplets were detected on the BioRad compared to the RainDance platform, while MAF remained similar across platforms and institutions. (**B**) Plasma-derived ctDNA analysis. Plasma-derived ctDNA was pre-amplified at CN via conventional PCR, using Assay A (right) and Assay B (left) primer/probe sets, prior to ddPCR analysis with Assay A across institutions. Speed vacuum concentration of samples was necessary to ensure preservation of input DNA in the requisite smaller sample input volume for the BioRad instrument. Superior test sensitivity was observed on the RainDance platform with both assays, while vacuum concentration increased observed MAF after Assay B ddPCR on the BioRad platform (t-test). % values = Mutation Allelic Frequency (MAF); * = Statistically significant difference in MAF (t-test*, p* < 0.05).

To optimize mutation detection in low starting [ctDNA], we isolated, concentrated and pre-amplified cfDNA from CSF (DMG-70-C) and plasma (DMG-168-P), then tested on RainDance (CN) and BioRad (UM, NU) platforms (Fig. 2, Supplementary Fig. 5). Importantly, we detected positive mutant droplets in all specimens, across all platforms and institutions, with no statistically significant difference in calculated MAF (Fig. 2, Supplementary Fig. 5). Speed vacuum-concentration of pre-amplified ctDNA further increased the number of positive droplets detected (Supplementary Fig. 5). We observed a statistically significant difference in calculated MAFs between Assay A and B ddPCR primer sets on the BioRad platform, due to poorer separation of positive and negative droplets with Assay A. Because the RainDance instrument can accommodate 12 µL sample input compared to 1-5 µL on BioRad, we found vacuum-concentration was necessary to ensure equivalent input [ctDNA] between platforms (Fig. 2B, Supplementary Fig. 5). There was no statistically significant difference in calculated MAFs between the samples analyzed on the RainDance versus speed-vacuum concentrated specimens on the BioRad instrument (Fig. 2).

### Cross-platform ddPCR validation with patient-matched samples

**Figure 3:**
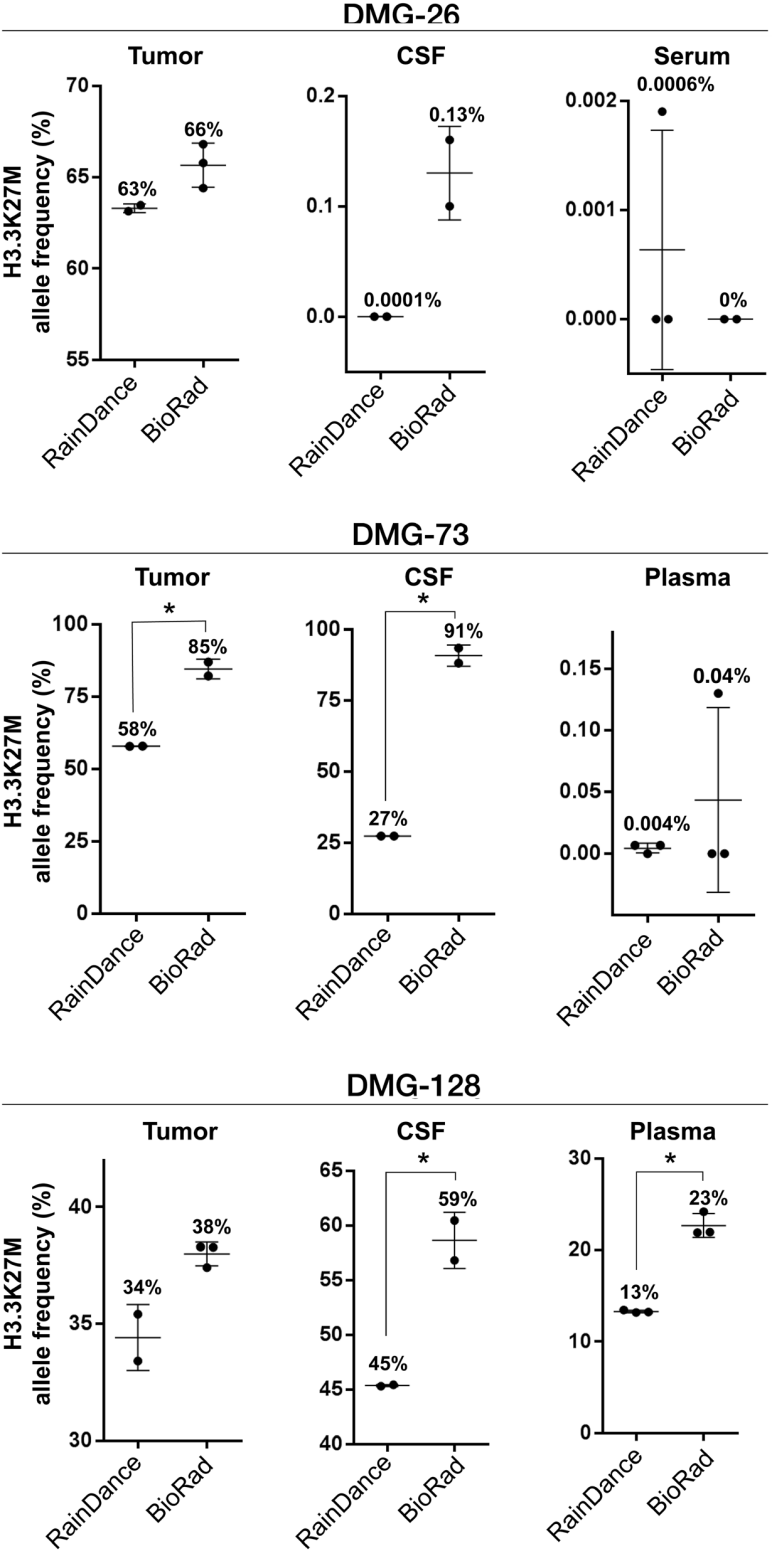
H3.3K27M mutant DNA is detectable in patient-matched tumor tissue, CSF and plasma or serum specimens. Patient-matched specimens from children with H3.3K27M mutant DMG (n = 3) were analyzed on the BioRad and RainDance platforms using Assay A. Mutant droplets were detected in all specimens across each subject tested, with greater MAF observed on the BioRad platform (t-test). Due to higher extracted [ctDNA] from DMG-128 CSF and plasma, samples were diluted 1:10 in DNA suspension buffer to prevent probe signal oversaturation. All other samples were diluted 1:5 as described. % values = Mutation Allelic Frequency (MAF); * = Statistically significant difference in MAF (t-test*, p* < 0.05).

To demonstrate reproducibility of our findings across ddPCR platforms, we analyzed matched tumor tissue, CSF and blood specimens from three patients with tissue-validated H3.3K27M mutant DMG (DMG-26, DMG-73, DMG-128, Fig. 3, Supplementary Fig. 6). Tissue gDNA and liquid specimen cfDNA were extracted, concentrated and pre-amplified at one site (CN) to minimize technical variability, and subsequently analyzed on RainDance (CN) and BioRad platforms (NU). Samples were analyzed using Assay A, given the greater demonstrated sensitivity and specificity of the assay (Fig. 1). The H3.3K27M mutation was detected in all samples tested, with fewer positive droplets in blood samples compared to CSF and tissue. MAF values were higher on BioRad, likely due to the noted differences in wildtype droplet separation using Assay A (Supplementary Fig. 6A).

### Cross-platform ddPCR validation by specimen origin

**Figure 4:**
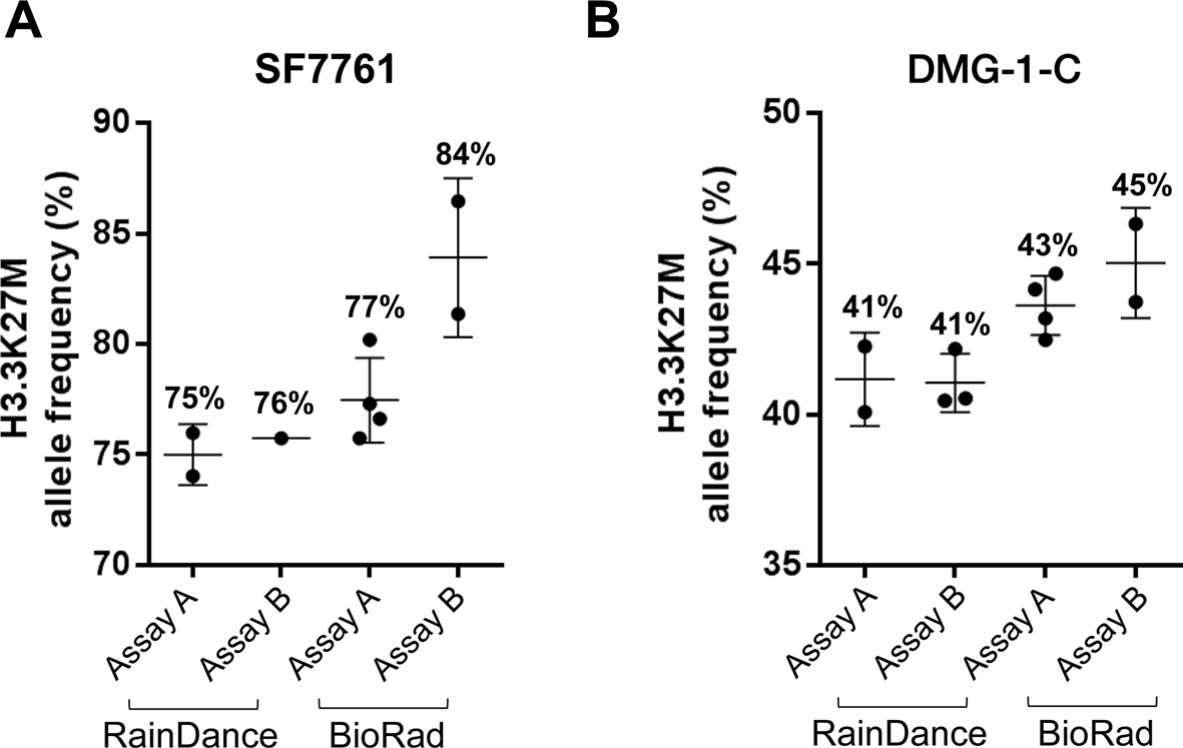
ddPCR results are consistent across institutions, irrespective of specimen preparation location. To determine the effects of location of sample DNA extraction and specimen shipment on ddPCR analysis results, DNA was extracted from (**A**) H3K27M mutant DMG CSF (DMG-1-C), and (**B**) DMG cells (SF 7761) at NU. ctDNA was pre-amplified using Assay C primers (NU), then analyzed on BioRad (NU) and RainDance (CN) platforms, with results compared to those from ctDNA extracted at CN. As with CN-prepared specimens, NU-prepared specimens yielded greater relative positive droplet counts in cell-derived ctDNA compared to CSF, and on RainDance compared to BioRad, with consistent droplet counts and MAFs between assays within a given platform. % values = Mutation Allelic Frequency (MAF).

All samples described thus far were prepared at CN prior to analysis. In these studies, comparable positive droplet counts were observed with both assays on a given platform, however a greater number of mutant droplets were consistently detected on RainDance. In order to determine whether this observed difference was due to DNA loss associated with sample shipping and handling or the ddPCR instrument itself, we also extracted and quantified DNA from tissue-validated H3.3K27M DMG mutant CSF (DMG-1-C) and cells (SF7761) at NU. Cell gDNA (2 ng) and CSF ctDNA (16 ng) were pre-amplified using Assay C primers, and paired aliquots were analyzed on BioRad (NU) or shipped for analysis on RainDance (CN). A greater number of positive droplets were consistently detected when using the RainDance instrument, regardless of the assay set used for ddPCR (Fig. 4). Importantly, there was no significant difference in calculated MAF between platforms regardless of sample origin and shipment (Fig. 4).

## Discussion

Pediatric DMG is universally fatal, with a high rate of histone H3 mutations. We previously demonstrated histone H3.3K27M mutation detection in DMG ctDNA from CSF and plasma, allowing longitudinal monitoring of changes in ctDNA level in liquid biopsy specimens without the need for repeated tumor tissue biopsy^17–19,23–25^. Given this potential clinical impact, we sought to optimize and validate our approach across multiple institutions and ddPCR platforms. This multi-institutional collaboration was critical, given the paucity of DMG specimens available for study and need for broadening clinical application across institutions. By pooling resources and testing different instruments, we optimized mutant droplet identification in specimens with very low starting [DNA], and identified technical nuances between systems. Even with pooled resources, the authors are aware of the limitations that specimen availability has placed on the strength of the study. However, we are able to show that ddPCR of liquid biopsy specimens could be reliably and robustly employed at multiple institutions, potentially making ctDNA-based mutation profiling a reality for more patients.

Overall, we found H3.3K27M mutation detection in blood specimens to be the most technically challenging due to very low starting [ctDNA]. To overcome this challenge, we employed vacuum-concentration of pre-amplified ctDNA, which increased test sensitivity without decreasing specificity (Fig. 2B, Supplementary Fig. 4), enabling target mutation detection in patient-matched tumor tissue, CSF and blood specimens (Fig. 3). It is important to note that few mutant droplets may be identified, with our previous study demonstrating a threshold of MAF > 0.001% as the lower limit of positive H3.3K27M detection in plasma (Supplementary Fig. 6). While our study included some samples that were obtained at autopsy to demonstrate proof-of-principle mutation detection, we note that much higher MAF was observed in postmortem relative to on-treatment plasma specimens (Fig. 3). Indeed, blood from patients with CNS tumors is known to harbor low levels of ctDNA relative to other tumor types, requiring exquisitely sensitive methods for mutation detection at diagnosis and during the course of treatment^26^. Despite this challenge, our work demonstrates the clinical feasibility of this approach. Future work may examine analysis of ctDNA level in plasma/serum samples following focused ultrasound-mediated blood brain barrier disruption, or possible changes in ctDNA availability in response to delivery of radiotherapy. These studies will be critical for identifying more sensitive time points for plasma ctDNA collection.

Overall, we noted differences in mutant droplet detection, and in mutant and wildtype cluster separation, when using different ddPCR instruments and primer/probe sets. These findings emphasize the need to (1) adjust ddPCR gating strategies based on droplet detection in negative and positive controls, and (2) evaluate the effect of amplicon length, optimum annealing temperature, and primer/probe length when designing ddPCR assays for enhanced specificity of mutation detection. As expected, primers that produce longer amplicons were more specific, but did result in poorer droplet separation. The LNA probes used for both Assays A and B were single-quenched and shorter in length, with the goal of maintaining high quenching frequency without requiring a double-quenched probe. However, probe length and sequence were dictated primarily by the ability to retain high binding specificity to H3.3K27M-mutant ctDNA.

Importantly, we show that ddPCR results are not hindered by the location of sample collection, processing, or analysis (Fig. 4), which is critical for developing protocols for clinical specimen analysis both locally and at collaborating institutions. Given that not all institutions may have access to ddPCR instruments, or may have access to different ddPCR instruments than their collaborators, the work presented here can guide protocol development for collaborative specimen sharing to inform clinical trials and improve patient treatment, without constraints imposed by geographical location and specimen access. Indeed, these findings support liquid biopsy as a rapid, cost-effective and minimally invasive method for ctDNA detection and monitoring^25^. The application of this precision medicine-based approach could help overcome current limitations for effective DMG treatment, including scarcity of tissue for molecular study. Further, detection of low-frequency tumor mutations using DNA from clinically accessible sources could enable validation of individualized, pre-clinical models for real time evaluation of patient response to specific therapies. Future work should be directed towards incorporating ddPCR of paired tissue and liquid specimens into prospective, longitudinal, cohort cross-sectional studies. Lastly, while our approach was optimized for *H3F3A* mutation detection in DMG, a similar platform could be tailored to investigate prognostic mutations in other cancers. As our knowledge of tumor molecular signatures expands, liquid biopsy may prove to be an increasingly valuable, broadly applicable tool in the armamentarium of precision medicine to improve patient care and clinical outcomes.

## Conclusion

We present an optimized, tissue-validated ddPCR workflow to identify and quantify H3.3K27M-mutant ctDNA in clinically accessible CSF and plasma specimens from DMG patients. Our results demonstrate that this approach is sensitive, specific, and reproducible across multiple institutions and technical platforms. This approach could therefore have significant utility for monitoring response to therapy and improving patient care.

## Supplementary Information

Supplementary Information.

## Abbreviations

CSF: Cerebrospinal fluid
CN: Children’s National Health System
CNS: Central nervous system
ctDNA: Circulating tumor DNA
ddPCR: Digital droplet PCR
gDNA: Genomic DNA
MAF: Mutant allelic frequency
NU: Northwestern University
DMG: Diffuse midline glioma
UM: University of Michigan
