# Supplementary material for: Standardization of the liquid biopsy for pediatric diffuse midline glioma using ddPCR
Source: Sci Rep. 2021 Mar 3;11:5098. doi: 10.1038/s41598-021-84513-1 (PMC7930089; doi:10.1038/s41598-021-84513-1)
Supplement: Supplementary file 1 — Supplementary Information. [file 41598_2021_84513_MOESM1_ESM.pdf]

Standardization of the liquid biopsy for pediatric diffuse midline glioma using ddPCR

Daphne Li MD, Erin R Bonner BA, Kyle Wierzbicki BS, Eshini Panditharatna PhD, Tina Huang MS, Rishi Lulla MD, Sabine Mueller MD, Carl Koschmann MD, Javad Nazarian PhD, Amanda M Saratsis MD

SUPPLEMENTARY MATERIAL

Supplemental Figures: 6
Supplemental Tables: 2

SUPPLEMENTARY FIGURE 1

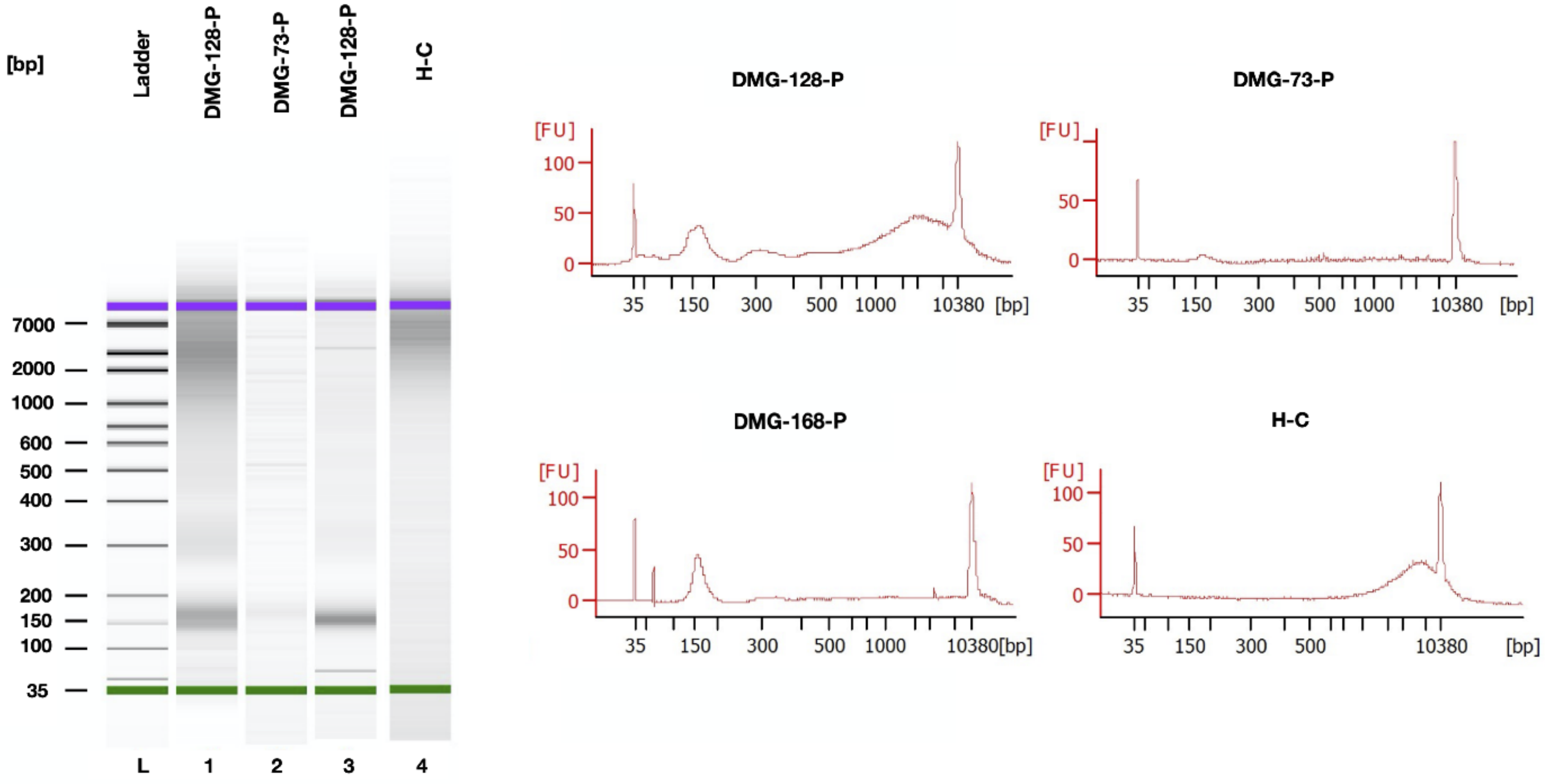

SUPPLEMENTARY FIGURE 2

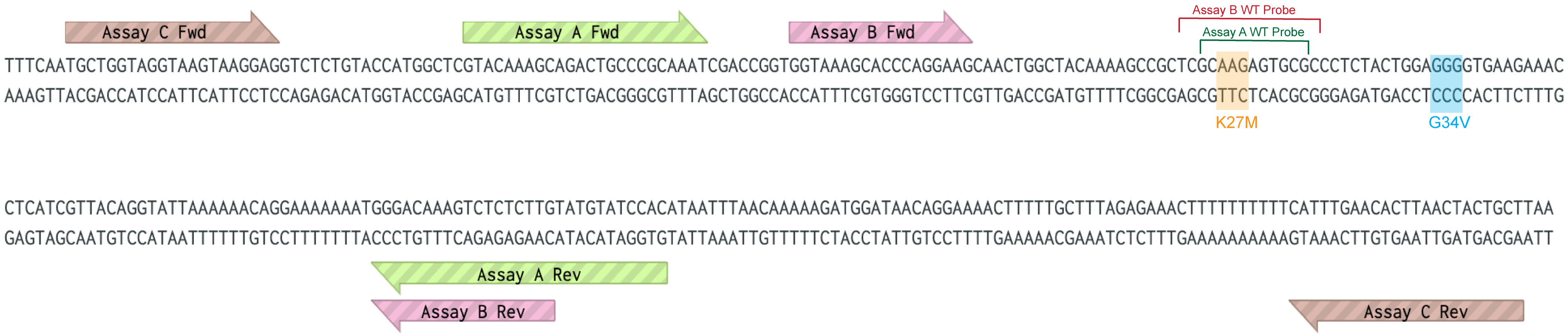

SUPPLEMENTARY FIGURE 3

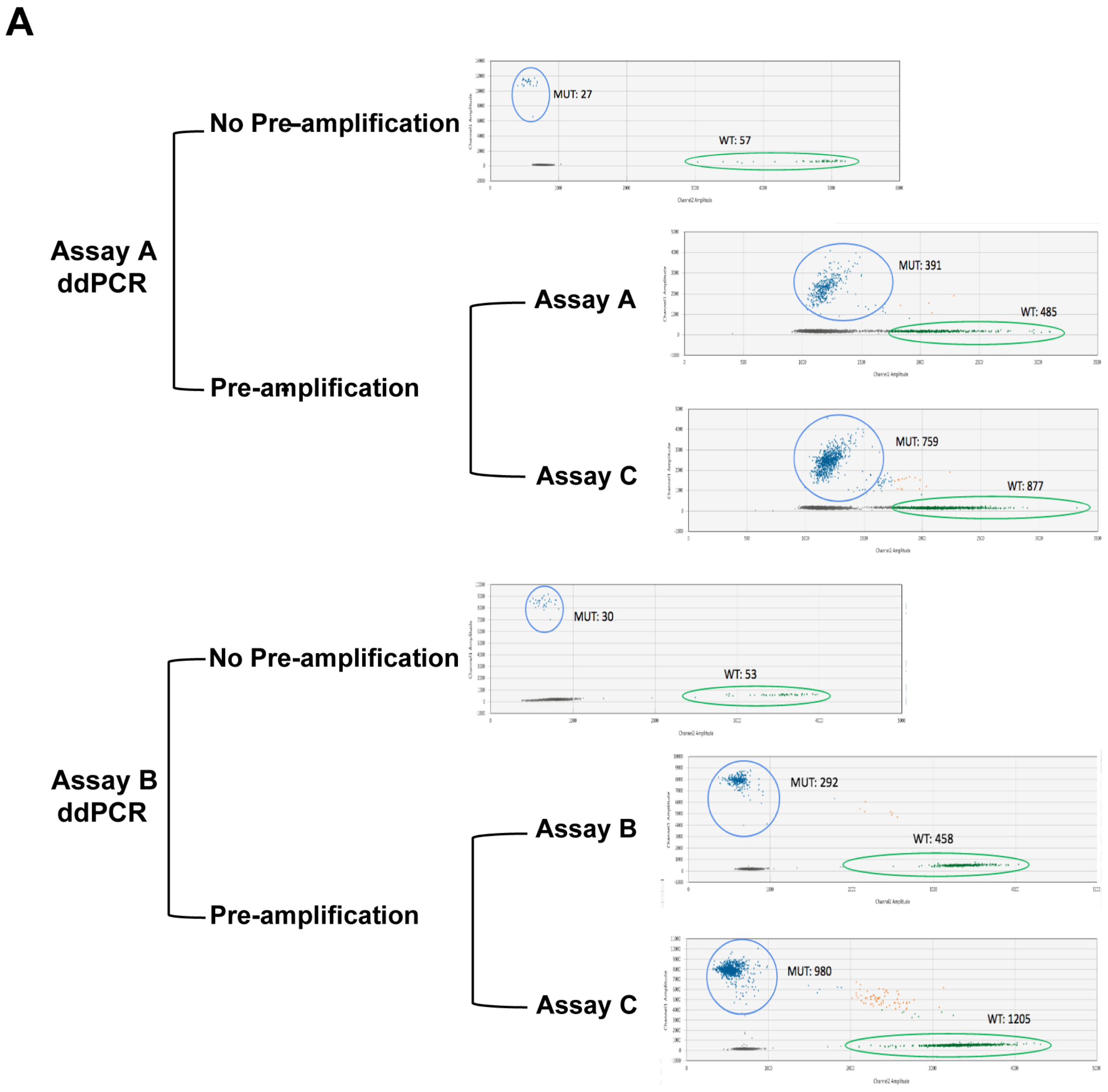

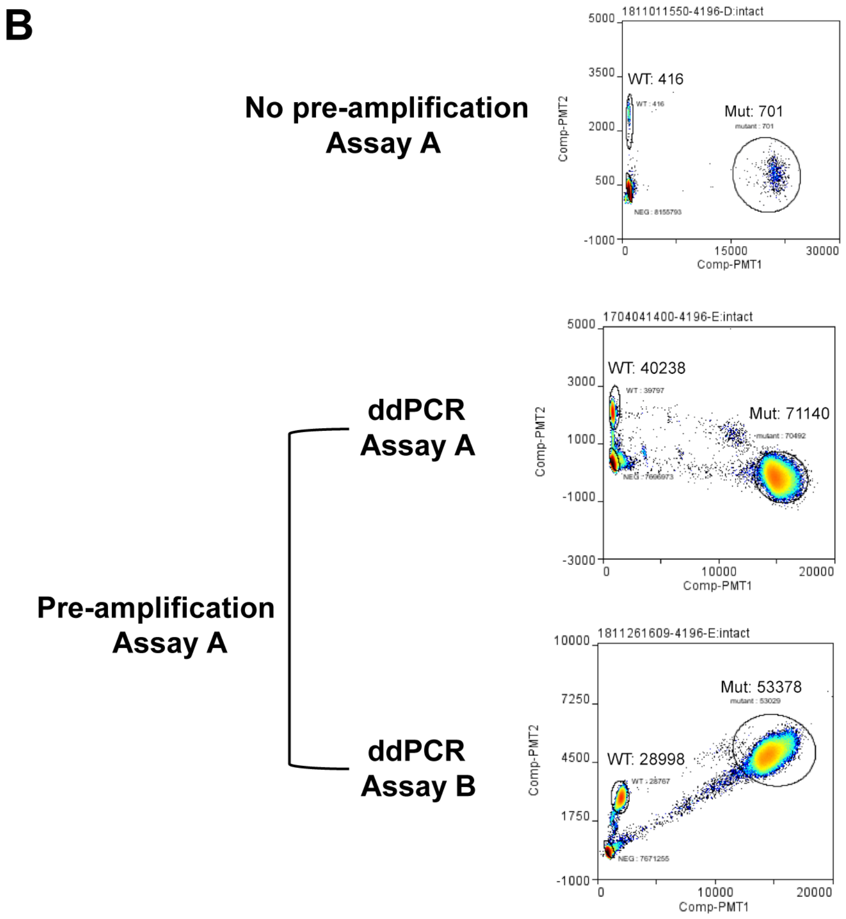
B
No pre-amplification
Assay A
Pre-amplification
Assay A
ddPCR
Assay A
ddPCR
Assay B
1811011550-4196-D:intact
WT: 416
Mut: 701
Comp-PMT2
Comp-PMT1
1704041400-4196-E:intact
WT: 40238
Mut: 71140
1811261609-4196-E:intact
Mut: 53378
WT: 28998

SUPPLEMENTARY FIGURE 4

A

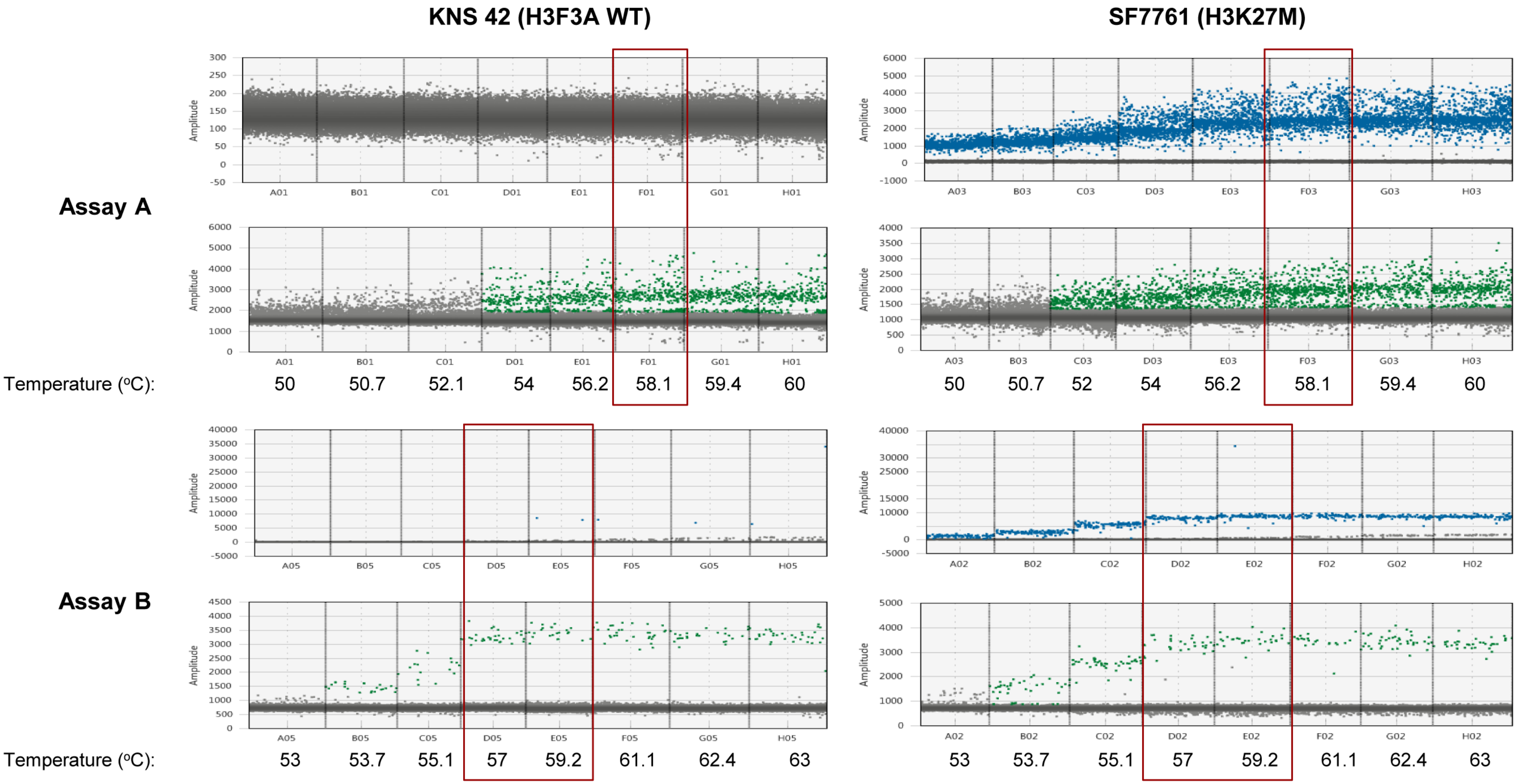

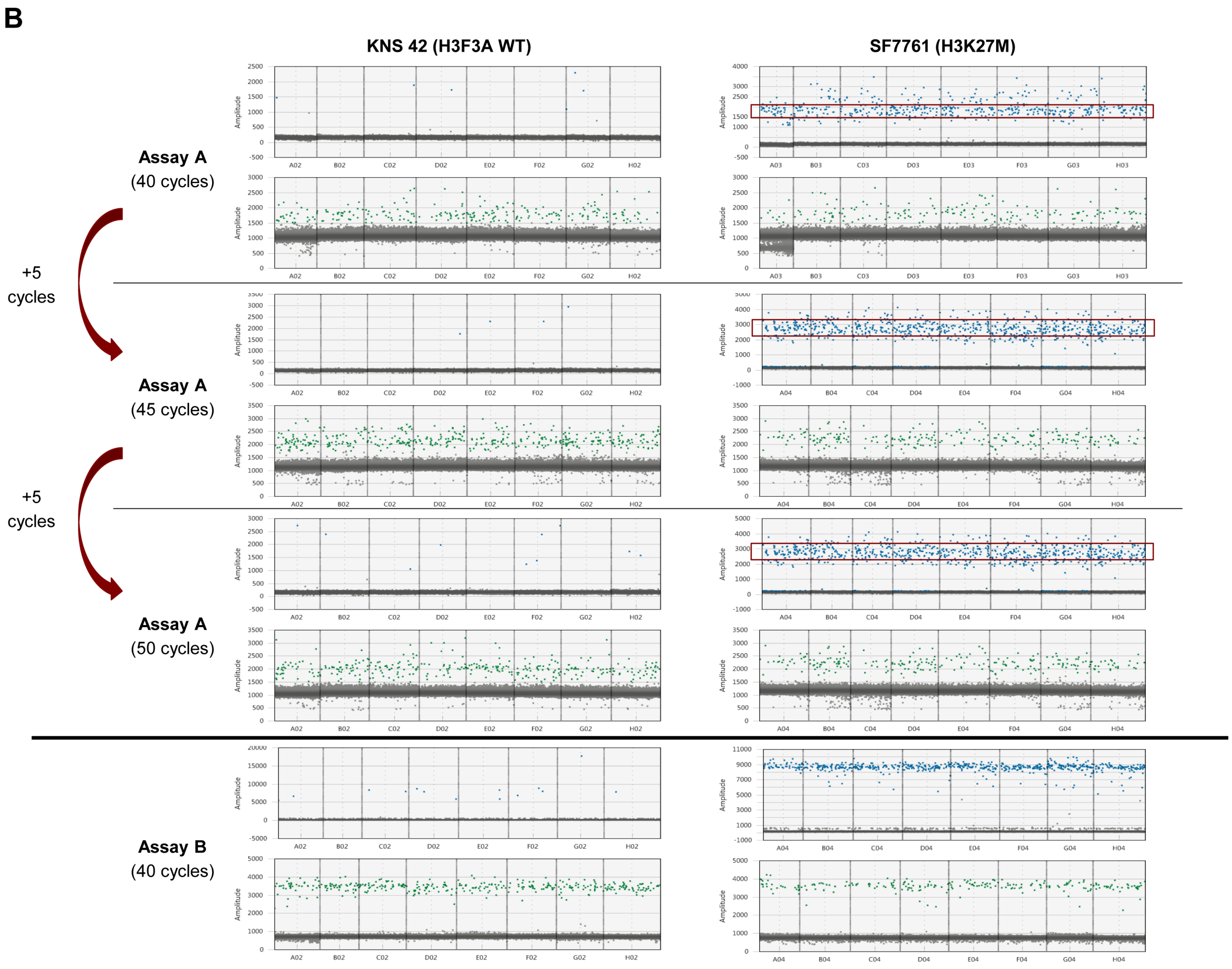

B
KNS 42 (H3F3A WT)
SF7761 (H3K27M)
Assay A
(40 cycles)
+5
cycles
Assay A
(45 cycles)
+5
cycles
Assay A
(50 cycles)
Assay B
(40 cycles)
Amplitude
A02
B02
C02
D02
E02
F02
G02
H02
A03
B03
C03
D03
E03
F03
G03
H03
A04
B04
C04
D04
E04
F04
G04
H04

SUPPLEMENTARY FIGURE 5

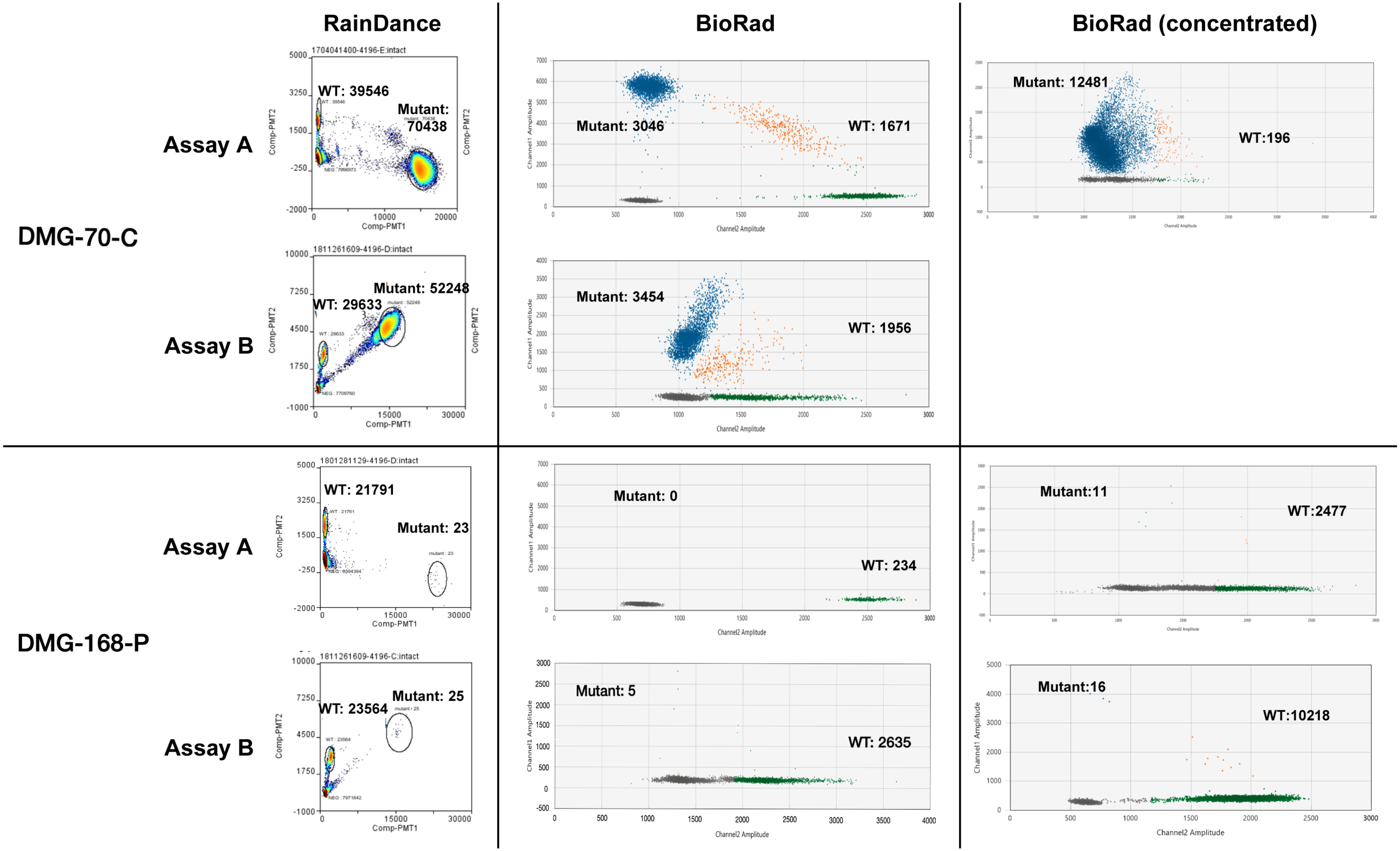

SUPPLEMENTARY FIGURE 6

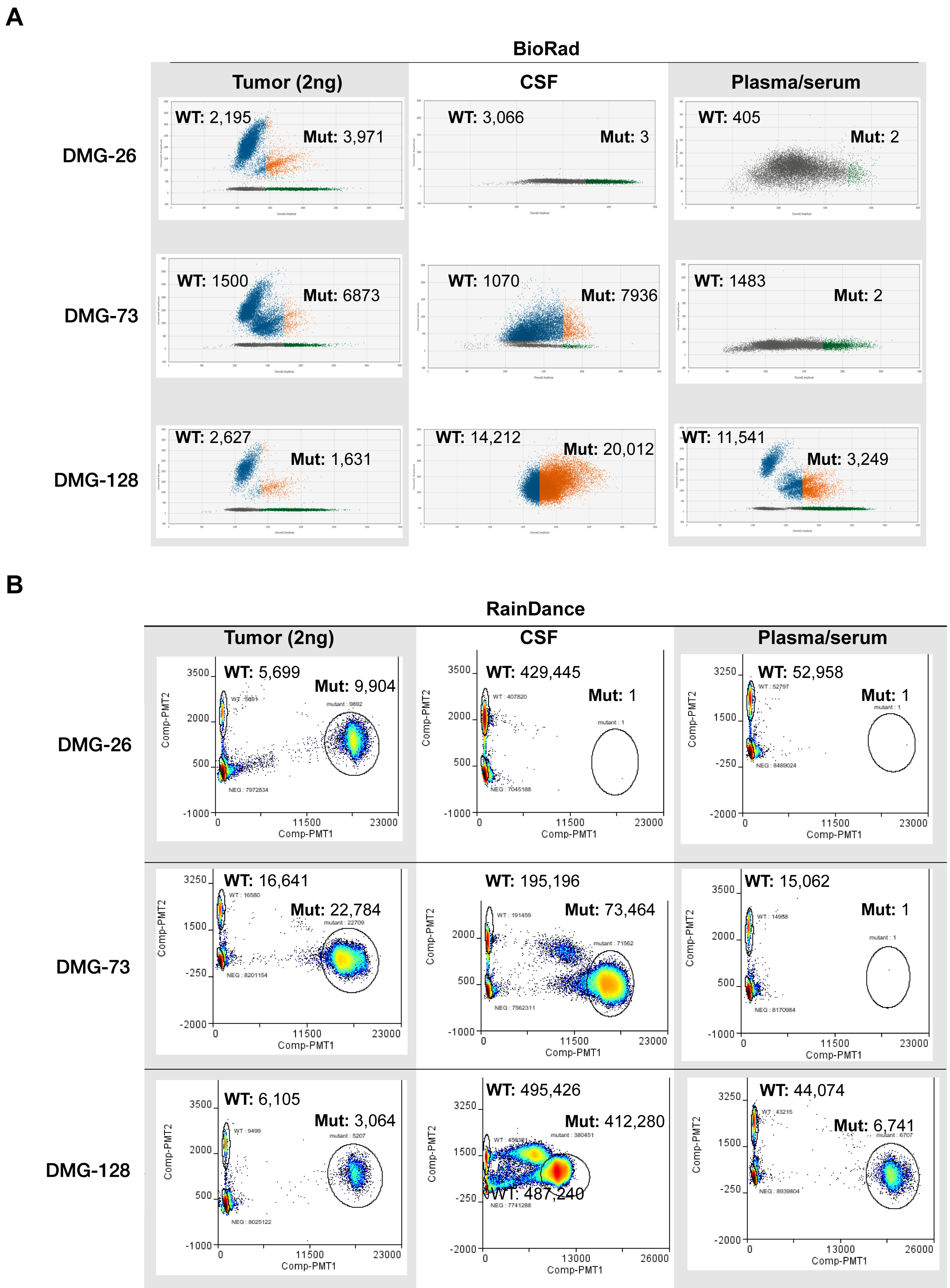

Supplementary Table 1. Biological Specimens used. All samples de-identified and labeled as follows in subsequent figures.

Sample	Type	Time point of Collection	Diagnosis	H3K27 status
**DMG-26**	Tumor	Postmortem	DMG	H3.3K27M Mutant
	CSF	Postmortem		
	Serum	During treatment		
**DMG-73**	Tumor	Postmortem	DMG	H3.3K27M Mutant
	CSF	Postmortem		
	Plasma	During treatment		
**DMG-128**	Tumor	Postmortem	DMG	H3.3K27M Mutant
	CSF	Postmortem		
	Plasma	Postmortem		
**DMG-51-T**	Tumor	Postmortem	DMG	H3.3K27M Mutant
**DMG-1-C**	CSF	During treatment	DMG	H3.3K27M Mutant
**DMG-70-C**	CSF	During treatment	DMG	H3.3K27M Mutant
**DMG-168-P**	Plasma	During treatment	DMG	H3.3K27M Mutant
**SF 7761**	Cell line	n/a	DMG	H3.3K27M Mutant
**KNS 42**	Cell line	n/a	Supratentorial GBM	Wild-Type
**H-C**	CSF	During treatment	Hydrocephalus	Wild-Type

Abbreviations: CSF = cerebrospinal fluid, DMG = diffuse midline glioma, GBM = glioblastoma multiforme

Supplementary Tables 2A-D. Cumulative ddPCR data generated through ddPCR analysis on BioRad Q200x and RainDance platforms. Abbreviations: avg = average, C = Cerebrospinal fluid, M = positive mutant droplet count, MAF = mutant-allelic frequency, P = plasma, P/A = pre-amplification, S = serum, stdev = standard deviation, T = tumor, V/C = vacuum concentration, WT = positive wildtype droplet count

A: Data generated for Figure 1A-C. ddPCR analysis of CSF ctDNA with and without pre-amplification

Figure	Specimen	Assay	Conditions	BioRad				RainDance			
				M	WT	MAF (%)	Avg MAF (%± stdev)	M	WT	MAF (%)	Avg MAF (%± stdev)
1A	Blank	A	No P/A	0	0	0	0±0				
				0	0	0					
				0	0	0					
	H-C			0	3	0	0±0				
				0	18	0					
				0	15	0					
	DMG-1-C			29	55	34.52	37.86±3.14				
				29	42	40.85					
				36	55	39.56					
				27	42	39.13					
				30	52	36.59					
				27	57	32.14					
				40	59	40.40					
				29	44	39.73					
	Blank		P/A with assay A	0	0	0	0±0				
				0	0	0					
				0	2	0					
	H-C			0	167	0	0±0				
				0	188	0					
				0	183	0					
				0	176	0					
	DMG-1-C			373	464	44.56	44.85±1.69				
				358	434	45.20					
				391	385	44.63					
				488	595	45.06					
				336	425	44.15					
				363	503	41.92					
				393	476	45.22					
				451	487	48.08					
	Blank		P/A with assay C	0	0	0	0±0				
				0	0	0					
				0	2	0					
	H-C			0	246	0	0±0				
				0	264	0					
				0	223	0					
				0	228	0					
	DMG-1-C			852	1029	45.30	46.66±0.77				
				897	976	47.89					
				781	892	46.68					
				893	1015	46.80					
				670	746	47.32					
				874	1018	46.19					
				857	979	46.68					
				759	877	46.39					
1B	Blank	B	No P/A	0	0	0	0±0				
				0	0	0					
				0	0	0					

				0	0	0					
	H-C			0<br>0<br>0<br>0	13<br>19<br>27<br>20	0<br>0<br>0<br>0	0±0				
	DMG-1-C			24<br>19<br>30<br>27<br>39<br>35<br>32<br>36	53<br>49<br>53<br>40<br>51<br>50<br>55<br>85	31.17<br>27.94<br>36.14<br>40.30<br>43.33<br>41.18<br>36.78<br>29.75	35.82±5.69				
	Blank		P/A with assay B	0<br>0<br>0<br>0	1<br>0<br>0<br>0	0<br>0<br>0<br>0	0±0				
	H-C			2<br>0<br>1	146<br>150<br>135	1.35<br>0<br>0.74	0.70±0.68				
	DMG-1-C			346<br>292<br>397<br>359<br>366<br>312<br>316<br>343	512<br>458<br>633<br>543<br>568<br>522<br>475<br>549`	40.33<br>38.93<br>38.54<br>39.80<br>39.19<br>37.41<br>39.95<br>38.45	39.08±0.95				
	Blank		P/A with assay C	0<br>0<br>0<br>0	0<br>0<br>0<br>0	0<br>0<br>0<br>0	0±0				
	H-C			0<br>0<br>0<br>1	111<br>95<br>104<br>101	0<br>0<br>0<br>0	0.25±0.49				
	DMG-1-C			818<br>799<br>884<br>980<br>825<br>778<br>906<br>942	1021<br>1069<br>1080<br>1025<br>1115<br>1013<br>1152<br>1238	44.48<br>42.77<br>45.01<br>48.88<br>42.53<br>43.44<br>44.02<br>43.21	44.29±2.03				
**1C**	DMG-70-C	A	No P/A					701<br>577<br>528	416<br>389<br>314	62.76<br>59.73<br>62.71	61.73±1.73
		A	P/A with assay A					70438<br>69026	39546<br>37542	64.04<br>64.83	64.44±0.56
		B	P/A with assay A					52589<br>52573<br>65434	29866<br>29095<br>36228	63.78<br>64.37<br>64.36	64.17±0.34

B: Data generated for Figure 2A and B. Optimization of ctDNA detection across technical platforms and institutions

				**BioRad**				**RainDance**			
**Figure**	**Specimen**	**Assay**	**Conditions**	**M**	**WT**	**MAF (%)**	**Avg MAF (%± stdev)**	**M**	**WT**	**MAF (%)**	**Avg MAF (%± stdev)**
**2A**	DMG-70-C	A	P/A, 12uL					70438	39546	64.04	64.44±0.56

								69026	37542	64.83	
		B	P/A, 12uL					52589<br>52573<br>65434	29866<br>29095<br>36228	63.78<br>64.37<br>64.36	64.17±0.34
		A	P/A, 1uL, no V/C	3046<br>3101	1671<br>1701	64.57<br>64.58	64.58±1.64E^{-5}				
		B	P/A, 1uL, no V/C	3454<br>2844	1956<br>1630	63.84<br>63.57	63.70±0.19				
**2B**	DMG-168-P	A	P/A, 12uL					23<br>24<br>20	21791<br>23166<br>21477	0.11<br>0.10<br>0.09	0.10±0.01
			P/A, 1uL, no V/C	1<br>13<br>5	1220<br>2931<br>2635	0.08<br>0.44<br>0.19	0.24±0.18				
			P/A, 1uL, V/C	10<br>11	2477<br>1362	0.40<br>0.80	0.60±0.28				
		B	P/A, 12uL					29<br>18<br>25	30637<br>22040<br>23598	0.095<br>0.082<br>0.106	0.09±0.01
			P/A, 1uL, no V/C	0<br>0	254<br>268	0<br>0	0±0				
			P/A, 1uL, V/C	16<br>13	10218<br>10448	0.16<br>0.12	0.14±0.03				

C: Data generated for Figure 3. H3.3K27M mutant DNA is detectable in patient-matched tumor tissue, CSF and plasma or serum specimens. All specimens were pre-amplified and analyzed using Assay A primers and/or probes.

		**BioRad (V/C)**				**RainDance**			
**Specimen**	**T/C/S/P**	**M**	**WT**	**MAF (%)**	**Avg MAF (%± stdev)**	**M**	**WT**	**MAF (%)**	**Avg MAF (%± stdev)**
**DMG-26**	**T**	3255<br>3843<br>3971	1618<br>1999<br>2195	66.80<br>65.78<br>64.40	65.66±1.20	9709<br>9904	5668<br>5699	63.14<br>63.47	63.31±2.37
	**C**	2<br>3	1215<br>3066	0.16<br>0.10	0.13±0.05	1<br>0	418466<br>429445	0.0002<br>0	0.0001±0
	**S**	0<br>0	405<br>14	0<br>0	0±0	0<br>1<br>0	51230<br>52958<br>50727	0<br>0.002<br>0	0.0006±0
**DMG-73**	**T**	6231<br>6873	937<br>1500	86.93<br>82.09	84.51±3.42	19724<br>22784	14351<br>16641	57.88<br>57.80	57.84±0.07
	**C**	4844<br>7936	344<br>1070	93.37<br>88.12	90.74±3.71	72042<br>73464	190741<br>195196	27.42<br>27.35	27.38±0.05
	**P**	0<br>2<br>0	518<br>1483<br>225	0<br>0.13<br>0	0.04±0.08	0<br>1<br>1	14683<br>14971<br>15062	0<br>0.007<br>0.007	0.004±0.004
**DMG-128**	**T**	1340<br>1437<br>1631	2242<br>2317<br>2627	37.41<br>38.28<br>38.30	38.00±0.51	3064<br>3046	6105<br>5554	33.42<br>35.42	34.42±1.42
	**C**	20012<br>18686	13094<br>14212	60.45<br>56.80	58.62±2.58	412280<br>396924	495426<br>479054	45.42<br>45.31	45.37±0.08
	**P**	2868<br>3159<br>3249	10213<br>9900<br>11541	21.92<br>24.19<br>21.97	22.69±1.30	6317<br>6741<br>6718	41542<br>43350<br>44074	13.20<br>13.46<br>13.23	13.29±0.14

D: Data generated for Figure 4A and B. ddPCR results are consistent across institutions, irrespective of specimen preparation location. All specimens were pre-amplified with Assay C primers.

Figure	Specimen	Assay	BioRad				RainDance			
			**M**	**WT**	**MAF (%)**	**Avg MAF (%± stdev)**	**M**	**WT**	**MAF (%)**	**Avg MAF (%± stdev)**
**4A**	SF7761	A	465<br>542<br>562<br>820	142<br>134<br>180<br>241	76.61<br>80.18<br>75.74<br>77.29	77.45±1.92	942<br>852	298<br>299	75.97<br>74.02	75.00±1.38
		B	134<br>144	21<br>33	86.45<br>81.36	83.90±3.60	933	299	75.73	75.73±0
**4B**	DMG-1-C	A	335<br>377<br>381<br>409	415<br>496<br>482<br>554	44.67<br>43.18<br>44.15<br>42.47	43.62±0.98	1142<br>986	1708<br>1347	40.07<br>42.26	41.17±1.55
		B	176<br>171	204<br>220	46.32<br>43.73	45.02±1.83	1013<br>1046<br>961	1389<br>1540<br>1410	42.17<br>40.45<br>40.53	41.05±0.97
